# Effectiveness of Improved Use of Chewing Gum During Capsule Endoscopy in Decreasing Gastric Transit Time: A Prospective Randomized Controlled Study

**DOI:** 10.3389/fmed.2021.605393

**Published:** 2021-02-15

**Authors:** Liang Huang, Yue Hu, Fang Chen, Shan Liu, Bin Lu

**Affiliations:** ^1^First Affiliated Hospital, Zhejiang Chinese Medical University, Hangzhou, China; ^2^Department of Gastroenterology, Hangzhou Red Cross Hospital, Hangzhou, China

**Keywords:** small bowel capsule endoscopy, chewing gum, gastric transit time, small bowel transit time, gastroscopy intervention

## Abstract

**Background/Aim:** Chewing gum throughout small bowel capsule endoscopy (SBCE) increases completion rates (CRs) but decreases small bowel transit time (SBTT) and diagnostic yield (DY). We determined the effects of chewing gum early during SBCE on gastric transit time (GTT), SBTT, CR, DY, and gastroscopy intervention.

**Methods:** We prospectively enrolled patients (ages 16–80 years) undergoing SBCE between January and June 2019. Patients were randomized to a chewing gum group (103 patients) and a control group (102 patients). Patients in the former group chewed one piece of gum for ~15 min every 30 min during the first hour of SBCE. Two gastroenterologists blinded to the study group examined the data.

**Results:** GTT was shorter in the chewing gum group (29.0 min, interquartile range: 17.0–52.0 min) than in the control group [42.5 min (23.25–60 min); *P* = 0.01]. SBTT was similar in the two groups [318.5 min (239.5–421.3 min) vs. 287.0 min (216.0–386.0 min); *P* = 0.08]. Gastroscopy rate was lower in the chewing gum group (15.53 vs. 32.35%, *P* = 0.005). CR (95.15 vs. 89.22%, *P* = 0.114) and DY (67.96 vs. 59.80%, *P* = 0.224) did not differ between the groups. The number of abnormal-lesion types detected per patient was higher in the chewing gum group [1.0 (0.0–2.0) vs. 2.0 (0.0–2.0); *P* = 0.049].

**Conclusions:** Chewing gum early during SBCE significantly reduced GTT and gastroscopy intervention, with no influence on SBTT (Trial number: NCT03815136).

## Introduction

Small bowel capsule endoscopy (SBCE) has greatly facilitated the screening, diagnosis, and monitoring of small bowel diseases (1, 2). SBCE has been shown to be efficacious for conditions such as celiac disease, iron-deficiency anemia, small bowel tumors, and familial polyposis syndromes (3). It is especially useful as a first-line investigation for obscure gastrointestinal bleeding (4). The two most vital quality indicators for SBCE are considered to be its diagnostic yield (DY) and completion rate (CR) (5, 6). However, several factors can affect the DY and CR of SBCE, including small bowel preparation, gastrointestinal transit time, and the battery life of the capsule endoscope (3). A gastric transit time (GTT) >45 min has been shown to be an independent risk factor for incomplete SBCE (7), while the DY of SBCE has been reported to be positively correlated with small bowel transit time (SBTT) (8). Moreover, gastric or esophageal retention of the capsule can prevent the endoscope from crossing the pylorus, prolonging GTT and even leading to a failure to reach the cecum within the available recording time. Furthermore, although the endoscopic placement of the capsule can be used to overcome this problem, this method increases patients' economic burden and discomfort (9).

The use of chewing gum throughout the SBCE examination simulates sham feeding, which shortens the GTT and/or SBTT and possibly increases the CR (10, 11). However, a decrease in SBTT may reduce the DY and therefore, the effectiveness of SBCE. We hypothesized that the limited use of chewing gum only in the first hour of the SBCE examination would shorten GTT but not SBTT and reduce gastroscopy intervention rate, and thereby improve the CR and DY of SBCE. The purpose of this study was to determine the effects of the limited use of chewing gum during SBCE on the GTT, SBTT, DY, CR and gastroscopy intervention of SBCE.

## Methods

### Study Design

This prospective, endoscopist-blind, randomized, controlled pilot study enrolled consecutive patients who were scheduled to undergo SBCE at the Endoscopy Center of the First Affiliated Hospital, Zhejiang Chinese Medical University, between January and June 2019. The institutional review board approved the study protocol and informed consent form (IRB number 2019-K-199-01). This study has been registered at www.ClinicalTrials.gov (NCT03815136).

### Study Participants

Patients aged 16–80 years who were scheduled to undergo SBCE and provided written informed consent were eligible to be included in this study. The exclusion criteria were as follows: previous abdominal surgery, diabetes mellitus, hyper- or hypothyroidism, use of prokinetics or narcotics within 5 days before SBCE, and refusal to participate in this study. In addition, patients with poor visibility on SBCE [visualization, <75% of the mucosa (12)] were considered to have received poor bowel preparation and were excluded from the study.

Patients were randomly assigned to a chewing gum group or a control group at the time of making the appointment for SBCE. We used envelopes containing computer-generated random numbers generated by one of the investigators (L.H.) who was responsible for keeping the randomization key locked until the last patient had been enrolled. The patients were instructed not to tell the endoscopist or the investigators (H.Y. and C.F.) about whether or not they used chewing gum before, during, or after the SBCE procedure.

### Study Procedures

All patients underwent identical bowel preparation prior to SBCE. The patients drank clear liquids for dinner on the day before capsule ingestion. On the day of the procedure, at 04:00–05:00 h, the patients drank two sachets of polyethylene glycol electrolyte powder dissolved in 2 L of water within a period of 2 h. Each sachet contained 59 g polyethylene glycol 4,000, 5.68 g sodium sulfate, 1.68 g sodium bicarbonate, 1.46 g sodium chloride, and 0.74 g potassium chloride (Hengkang Pharmaceutical Co, Jiangxi, China). In addition, the patients fasted overnight for at least 8 h before undergoing SBCE.

All examinations were commenced between 8:00 and 10:00 h. We used PillCam SB2 (Medtronic, Minnesota, America) for the examinations. Patients allocated to the chewing gum group were instructed to chew a piece of sugarless gum (Wrigley's Extra Sugar-Free Gum) for ~15 min every 30 min during the first hour of the SBCE examination. Thus, each patient in this group chewed a total of 2 pieces of gum. The intervention was timed using an alarm. The patients allocated to the control group were not given chewing gum and were asked to refrain from doing a chewing movement.

All patients returned for review ~60 min later for real-time confirmation of whether the capsule camera had reached the small bowel. If it had not, the patient underwent gastric endoscopic placement of the capsule. The intake of solid foods was permitted 8 h after capsule ingestion. All patients returned to the SBCE recorder after 24 h of examination.

### Outcomes

The primary outcomes were GTT and SBTT. GTT was defined as the interval between the first gastric and first duodenal images, while SBTT was defined as the interval between the first duodenal and first cecal images. The secondary outcomes were DY and CR. DY was defined as the rate of positive findings (diagnostic or suspicious) on SBCE examination, and CR was defined as the rate of complete recording, as indicated by the camera entering the cecum within the battery time. Capsule endoscopic finding per patient was sorted according to the following categories: normal variants (lymphangiectasia and lymphatic follicular hyperplasia) and abnormal lesions [ulcer, polyp, bleeding, vascular malformation, stenosis, diverticulum, parasite, tumor, and inflammation, which included inflammatory changes observed in the intestine during endoscopy (13), such as erosion, Crohn's disease, eosinophilic enteritis, intestinal tuberculosis, and radiation enteritis]. We also assessed the intervention rate, which was defined as the rate of endoscopic capsule placement in the duodenum when the GTT was over 60 min (9).

### Sample-Size Calculation

Prior to the study, we calculated the sample size required based on our preliminary results, namely GTT (data shown in Supplementary Material). We determined that at least 182 patients were needed to detect significant differences on two-tailed tests with a 0.05 alpha level and 80% power. In our experience, approximately 10% of patients cancel their SBCE appointments or have bad bowel preparation. Thus, a total sample size of 203 patients was estimated to be sufficient.

### Statistical Analysis

Descriptive statistics were used for the baseline characteristics of the participants. Specifically, continuous variables with normal distribution were expressed as mean and SD (age and body-mass index), and compared using the Student *t*-test, while categorical variables were expressed as percentages and counts (gender and indication for capsule endoscopy), and compared using the χ^2^-test for categorical variables.

Between-group differences in outcomes were analyzed using the χ^2^-test in the case of categorical variables (DY, CR, and intervention rate) and the Mann–Whitney *U*-test of variance in the case of continuous variables with non-normal distribution (GTT, SBTT, and abnormal lesions per patient). GTT, SBTT, and abnormal lesion type per patient were expressed as medians and interquartile ranges (25th−75th percentiles). The hazard ratios for complete viewing of the stomach and small bowel in the control and chewing gum groups were assessed using Kaplan-Meier analysis (Log-rank). The statistical analysis was conducted using SPSS for Windows, version 23.0. All reported *P*-values were two-sided, and confidence intervals (CIs) were at the 95% level. Differences with *P* < 0.05 were considered statistically significant.

## Results

### General Information

**Figure 1:**
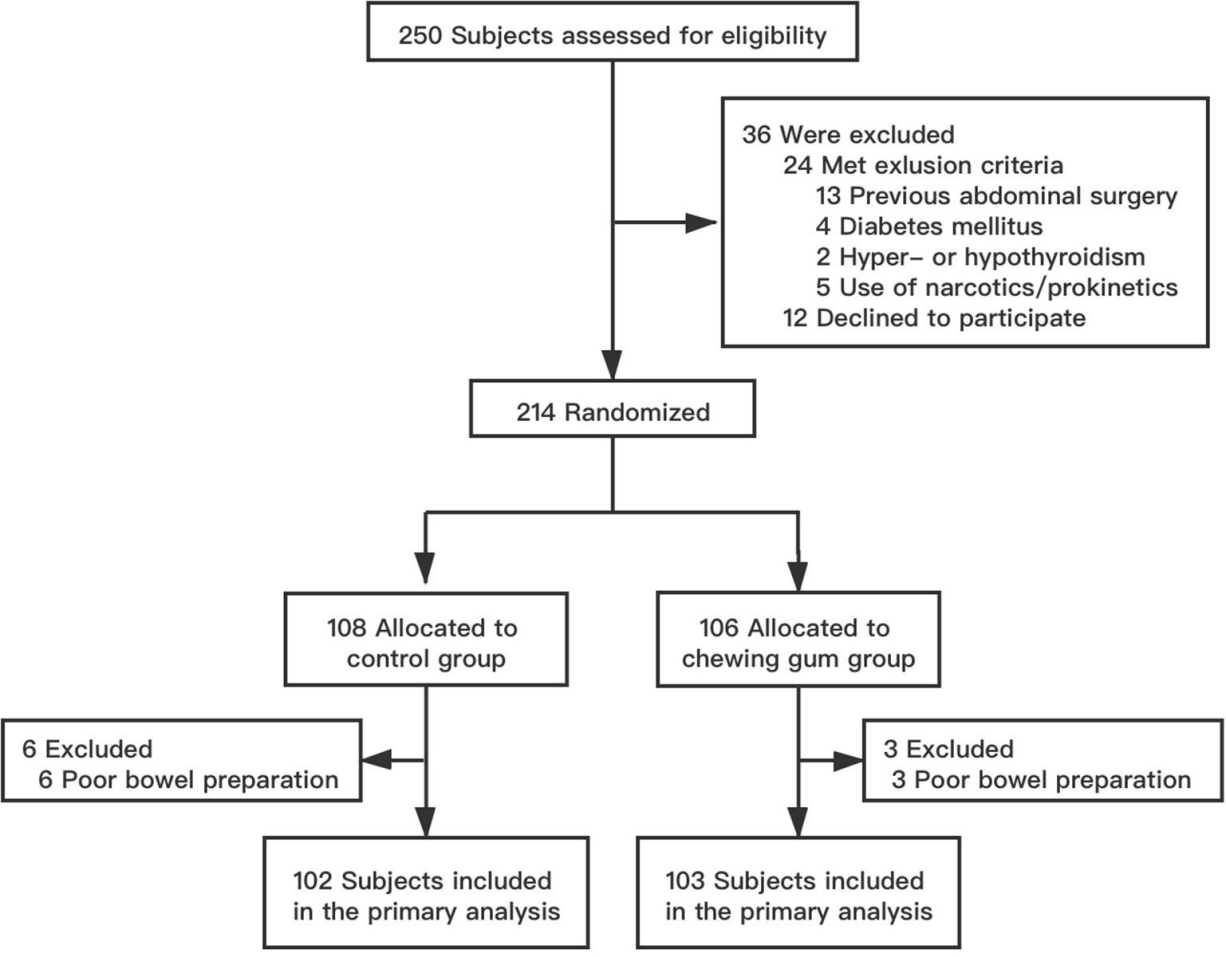
Flowchart of the study.

Between January and June 2019, a total of 250 patients aged 16–80 years were scheduled to undergo SBCE in our institution. Of these, 36 patients were excluded because they either did not meet the selection criteria (24 patients) or refused to participate in the study (12 patients). The remaining 214 patients were randomized to the control group (108 patients) and the chewing gum group (106 patients). Six patients from the control group and three patients from the chewing gum group were unable to undergo SBCE at the scheduled time because of poor bowel preparation (*P* = 0.514). Thus, the final analysis involved 102 subjects in the control group and 103 subjects in the chewing gum group. A flow chart of subject selection is shown in Figure 1. The baseline clinical characteristics were comparable between the two study groups (Table 1).

**Table 1:** Characteristics of the study subjects.

**Parameter**	**Control group**	**Chewing gum group**	***P*-value**
	**(*n* = 102)**	**(*n* = 103)**	
Age, yrs, mean ± SD	45.00 ± 15.60	47.10 ± 16.30	0.347
Gender			0.953
Female, *n* (%)	43 (42.16)	43 (41.75)	
Male, *n* (%)	59 (57.84)	60 (58.25)	
BMI, kg/m^2^, mean ± SD	21.63 ± 2.94	21.92 ± 3.85	0.536
Indication for capsule endoscopy			0.245
Screening, *n* (%)	33 (32.35)	26 (25.24)	
Diagnostic, *n* (%)	65 (63.73)	68 (66.02)	
Surveillance, *n* (%)	4 (3.92)	9 (8.73)	

*BMI, body-mass index*.

*P-value: Student t-test for continuous variables and χ^2^-test for categorical data*.

### Outcomes

**Figure 2:**
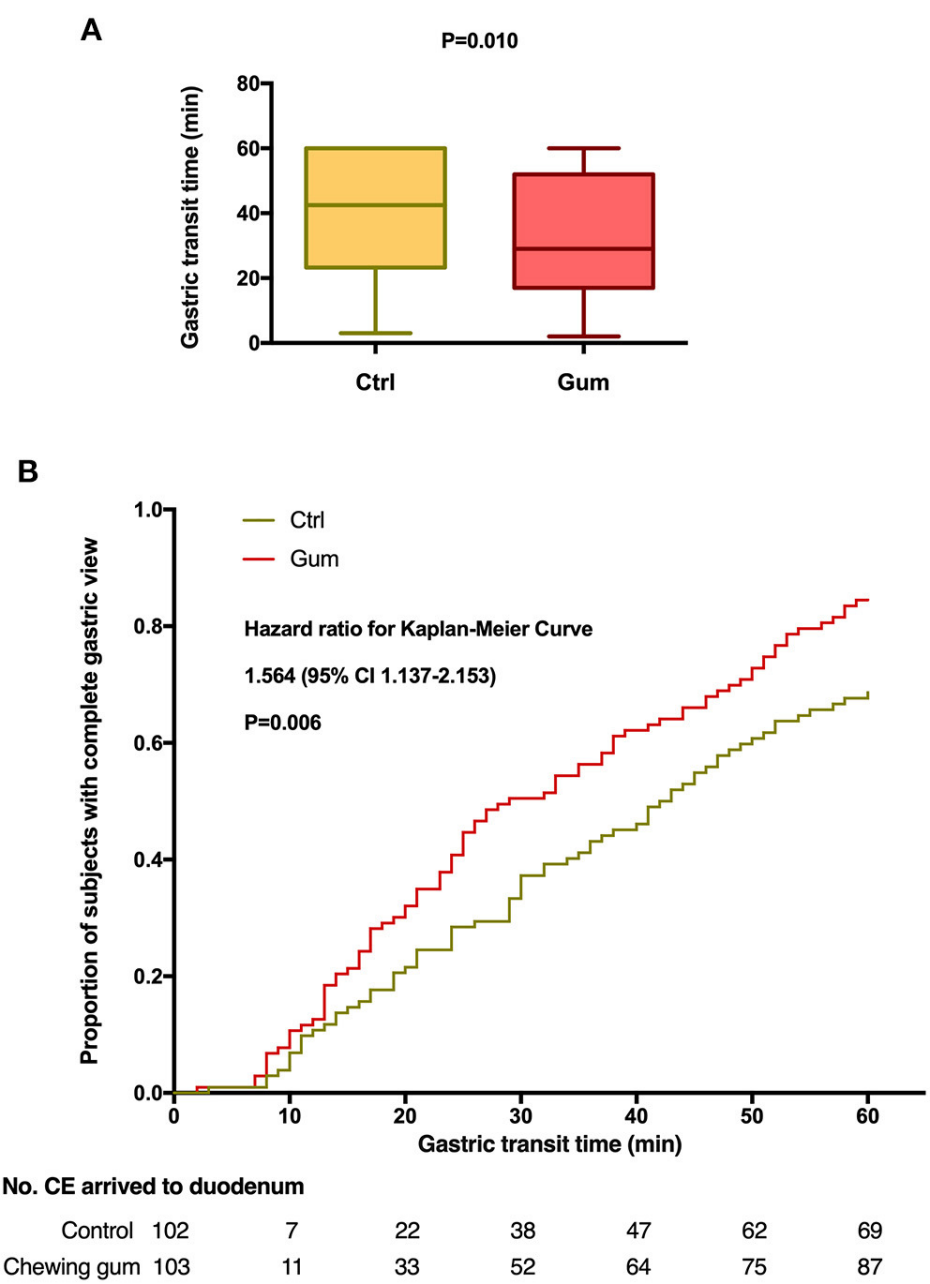
Gastric transit time during capsule endoscopy in the chewing gum group and control group. **(A)** A boxplot with medians and quartiles; **(B)** Kaplan–Meier curves for time to complete gastric view.

**Figure 3:**
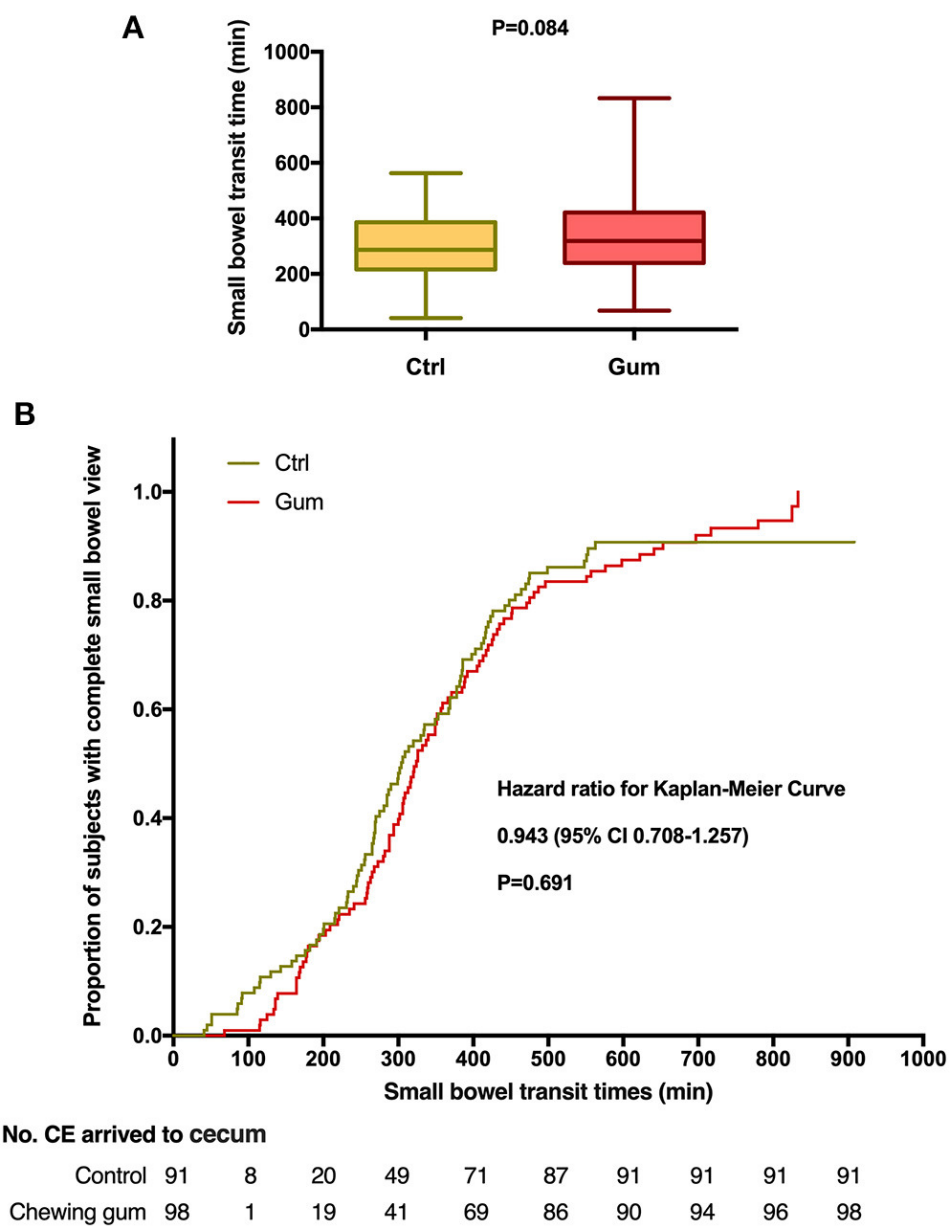
Small bowel transit time during capsule endoscopy in the chewing gum group and control group. **(A)** A boxplot with medians and quartiles; **(B)** Kaplan–Meier curves for time to complete small bowel view.

The outcomes of the study have been summarized in Table 2. The median GTT was significantly lower in the chewing gum group (29.0 min) than in the control group [42.5 min; Kaplan–Meier: hazard ratio, 1.564; 95% confidence interval (CI), 1.137–2.153; *P* = 0.006; Figure 2]. In contrast, the median SBTT did not differ between the chewing gum group (318.5 min) and control group (287.0 min; Kaplan–Meier: hazard ratio, 0.943; 95% CI, 0.708–1.257; *P* = 0.6913; Figure 3).

**Table 2:** Effects of chewing gum on the outcomes of capsule endoscopy examination.

**Parameter**	**Control group**	**Chewing gum group**	***P*-value**
	**(*n* = 102)**	**(*n* = 103)**	
GTT, min, median (IQR)	42.5 (23.25, 60.00)	29.0 (17.00, 52.00)	0.010
SBTT, min, median (IQR)	287.0 (216.00, 386.00)	318.5 (239.50, 421.30)	0.084
Completion rate, *n* (%)	91 (89.22)	98 (95.15)	0.114
Diagnostic yield, *n* (%)	61 (59.80)	70 (67.96)	0.224
Abnormal lesions type, *n*, median (IQR)	1 (0.00, 2.00)	2 (0.00, 2.00)	0.049
Intervention rate, *n* (%)	33 (32.35)	16 (15.53)	0.005

*GTT, gastric transit time; SBTT, small bowel transit time; IQR, interquartile range*.

*P-value: Mann–Whitney U-test of variance for alphanumeric variables and χ^2^-test for categorical data*.

The DY and CR also did not significantly differ between the chewing gum and control groups (*P* = 0.224 and *P* = 0.114, respectively). The number of abnormal small bowel-lesion types detected per patient was higher in the chewing gum group than in the control group (*P* = 0.049). The intervention (gastroscopy) rate was significantly lower in the chewing gum group than in the control group (15.53 vs. 32.35%, *P* = 0.005).

### Findings and Incomplete Examinations

The most frequent positive result on SBCE was gastrointestinal ulcer (control, 43.13%; chewing gum, 44.66%), followed by gastrointestinal inflammation (control, 21.57%; chewing gum, 35.92%) and small bowel stenosis (control, 9.80%; chewing gum, 15.53%). Other SBCE findings included small bowel bleeding, small bowel vascular malformation, polyp syndrome, small bowel tumor, intestinal parasites, and intestinal diverticulum (Table 3).

**Table 3:** Findings of capsule endoscopy examination.

**Parameter**	**Control group**	**Chewing gum group**
	**(*n* = 102)**	**(*n* = 103)**
**Small-bowel abnormal lesions**		
Ulcer, *n* (%)	44 (43.13)	46 (44.66)
Inflammation, *n* (%)	22 (21.57)	37 (35.92)
Polyps, *n* (%)	7 (6.86)	4 (3.88)
Diverticulum, *n* (%)	None	1 (0.97)
Parasite, *n* (%)	1 (0.98)	5 (4.85)
Bleeding, *n* (%)	4 (3.92)	8 (7.77)
Vascular malformation, *n* (%)	2 (1.96)	9 (8.74)
Tumor, *n* (%)	2 (1.96)	1 (0.97)
Stenosis, *n* (%)	10 (9.80)	16 (15.53)
**Normal variants**		
Lymphangiectasia, *n* (%)	12 (11.76)	10 (9.71)
Lymphatic follicular hyperplasia, *n* (%)	7 (6.86)	4 (3.88)
Normal, *n* (%)	34 (33.33)	25 (24.27)

SBCE could not be completed for 11 patients in the control group and five patients in the chewing gum group because of either capsule retention (due to carcinoma and ulcer) or battery power outage (Table 4). Among the nine patients with incomplete SBCE due to capsule retention, the following interventions were carried out: in one patient, the capsule was excreted naturally after 7 days without any therapy; in two patients diagnosed with small intestinal carcinoma, the capsule was retrieved during surgical intervention; in two patients, the capsule was passed after medical therapy (at 1 month and 67 days); and in three patients, the capsule was retrieved using double-balloon enteroscopy.

**Table 4:** Reasons for non-completion of capsule endoscopy examination.

**Parameter**	**Control group**	**Chewing gum group**
	**(*n* = 11)**	**(*n* = 5)**
Battery power outage, *n* (%)	5 (45.45)	2 (40.00)
Capsule retention, *n* (%)	6 (54.55)	3 (60.00)
Small intestinal stenosis, *n* (%)	6 (54.55)	3 (60.00)
Carcinoma, *n* (%)	1 (9.09)	1 (20.00)
Ulcer, *n* (%)	5 (45.45)	2 (40.00)

## Discussion

The present study revealed that patients who chewed two pieces of gum during the first hour of the SBCE procedure had a significantly shorter GTT than control subjects who did no chewing movements during SBCE. The intervention (gastroscopy) rate was also significantly lower in the chewing gum group than in the control group. However, the SBTT, DY, and CR did not differ between the two groups. Interestingly, although the DY did not significantly differ between the two study groups, further analysis showed that the number of abnormal lesion type detected per patient was higher in the chewing gum group than in the control group.

Prokinetic drugs and metoclopramide have been used to shorten the GTT and/or SBTT and increase the CR of SBCE (14–16); however, these agents promote gastric as well as small intestinal movement throughout the examination period, and thus, limit the DY (4). Chewing gum is a safe, convenient, and inexpensive method of shortening the GTT via activation of vagal cholinergic stimulation of the bowel by simulating sham feeding (17, 18). However, studies on the effects of chewing gum on GTT, SBTT, and CR have reported mixed findings (10, 11, 19). Consistent with our study, Apostolopoulos et al. (11) reported that the use of chewing gum during SBCE shortened the GTT. However, all of these previous studies used chewing gum throughout the SBCE examination, leading to a decrease in the SBTT and therefore the DY (8, 20). In contrast, chewing gum use was limited to the first hour of SBCE in our study, and we found that although the SBTT was longer in the chewing gum group than in the control group, the difference was not statistically significant. It is reasonable that chewing gum during the first hour of intervention did not increase the intestinal motility.

In our study, the CR did not differ between the two study groups. However, it should be noted that in most patients, the reason for the non-completion of the examination was capsule retention due to the presence of a lesion in the small intestine. Shortening the GTT through chewing gum use is not expected to have an effect on the completion of the examination in such patients. Moreover, compared to the control group, the chewing gum group showed a higher number of abnormal lesions categories for per patient, which may theoretically lead to an increase in capsule retention rates, though no statistical difference was found in the present study. It should however be noted that the DY did not significantly differ between the chewing gum and control groups. There are several potential reasons for the lack of an increase in the SBTT, DY, and CR in our study. First, our study included both participants with normal findings on SBCE as well as participants with diverse clinical conditions; this could have limited the DY after randomization. Second, intestinal motility may have differed between the two study groups, which may have contributed to the lack of significant differences in the above parameters. Third, the sample size was small, and was calculated based on GTT before this trial, which might explain the negative results. Finally, the gastroscopy intervention in the first hour would have reduced the differences in SBTT, DY, and CR between the two study groups by reducing the original GTT.

In this study, the gastroscopy intervention rate was significantly lower in the chewing gum group than in the control group. This indicated that the use of chewing gum during the first hour of the examination could enhance gastrointestinal motility and accelerate the transit of the capsule endoscope through the stomach. In our clinic, we usually check the position of the capsule endoscope at 30 min after swallowing, and perform some intervention (mainly gastroscopy) if the capsule fails to pass through the stomach at 1 h after swallowing. The decrease in the gastroscopy rate indicated that the use of chewing gum early during the procedure could reduce human intervention during SBCE, especially in terms of gastroscopy, which can cause discomfort to patients. In addition, Buijs et al. (21) reported that chewing two pieces of sugar-free chewing gum after the capsule left the stomach could increase the excretion rate (not significantly), which might improve the bowel-cleansing quality. However, we compared the rate of poor bowel preparation between the two groups, and found no significant difference in this rate (5.56 vs. 2.83%, *P* = 0.514). In our study, the chewing gum intervention was mainly restricted to the period when the capsule was in the stomach in the first hour of the examination, which might explain the relatively limited effect on bowel preparation. Previous studies indicated that capsule endoscopy has a good diagnostic yield rate in iron-deficiency anemia patients, about 47% (22). In particular, more vascular (31 vs. 22.6%, *P* = 0.007), inflammatory (17.8 vs. 11.3%, *P* = 0.009), and mass/tumor (7.95 vs. 2.25%, *P* = 0.0001) lesions were detected with SBCE (22). Contaldo et al. (23) showed that VCE could reveal the source of obscure-occult bleeding in a high percentage of unexplained iron-deficiency anemia. However, our result did not find the validity of SBCE in the investigation of patients with IDA and negative findings on a previous diagnostic workup.

The current study has a few limitations. First, this was a single-center study, which may have affected the outcomes due to the bias of the collected data. Second, there is no true gold standard test against which SBCE may be compared, and therefore, there may be an underlying rate of missed cases that cannot be assessed. However, as the repeatability of SBCE in the same patients may be objective and reliable, and this can be used to reduce bias to a certain extent in future studies. Finally, the higher detection rate of abnormal-lesion types in the chewing gum group as compared to the control group was on the borderline of statistical significance (*P* = 0.049). We therefore performed logistic regression analysis to evaluate the effects of age, sex, body-mass index, and number of abnormal-lesion types. Among these factors, only the number of abnormal-lesion types was found to be significant (*P* = 0.034, odds ratio = 1.358, 95% confidence interval: 1.024–1.801). This result lends further support to the study findings. In the future, more high-quality, large-scale studies will help overcome these limitations and draw more solid conclusions.

In summary, the limited use of chewing gum during the first hour of SBCE significantly reduced the GTT but not the SBTT. The use of chewing gum did not reduce the CR of SBCE and significantly reduced the gastroscopy intervention rate. We believe that the use of chewing gum during the first hour of SBCE might improve the detection of abnormal lesions, as the SBTT remains unaltered during the procedure.

## Data Availability Statement

The original contributions presented in the study are included in the article/Supplementary Material, further inquiries can be directed to the corresponding author/s.

## Ethics Statement

The studies involving human participants were reviewed and approved by The institutional review board approved the study protocol and informed consent form (the First Affiliated Hospital, Zhejiang Chinese Medical University;IRB number 2019-K-199-01). Written informed consent to participate in this study was provided by the participants' legal guardian/next of kin.

## Author Contributions

BL designed and supervised the study, including all data collection and analysis. LH, YH, and FC performed most of the investigations, including data collection and analysis, and wrote the manuscript. SL provided guidance on data analysis. All authors have read and approved the manuscript.

## Conflict of Interest

The authors declare that the research was conducted in the absence of any commercial or financial relationships that could be construed as a potential conflict of interest.

## Supplementary Material

Excel Sheet 1 Original data.

Excel Sheet 2 Original data of pre-experiment.
