# Corrigendum: Effectiveness of Improved Use of Chewing Gum During Capsule Endoscopy in Decreasing Gastric Transit Time: A Prospective Randomized Controlled Study

**DOI:** 10.3389/fmed.2021.805642

**Published:** 2022-01-24

**Authors:** Liang Huang, Yue Hu, Fang Chen, Shan Liu, Bin Lu

**Affiliations:** ^1^First Affiliated Hospital, Zhejiang Chinese Medical University, Hangzhou, China; ^2^Department of Gastroenterology, Hangzhou Red Cross Hospital, Hangzhou, China

**Keywords:** small bowel capsule endoscopy, chewing gum, gastric transit time, small bowel transit time, gastroscopy intervention

In the original article, there was an error. “19.0 min” should be “29.0 min” in the **Abstract**.

A correction has been made to **Abstract**, **Results**, Paragraph One:

**Results:** GTT was shorter in the chewing gum group (29.0 min, interquartile range: 17.0–52.0 min) than in the control group [42.5 min (23.25–60 min); *P* = 0.01].

In the original article, there was an error. “DY (66.02 vs. 59.80%, *P* = 0.359)” should be “DY (67.96 vs. 59.80%, *P* = 0.224)” in **Abstract**.

A correction has been made to **Abstract, Results**, Paragraph One:

CR (95.15 vs. 89.22%, *P* = 0.114) and DY (67.96 vs. 59.80%, *P* = 0.224) did not differ between the groups.

In the original article, there was an error. “*P* = 0.359” should be “*P* = 0.224” in the **Results** section.

A correction has been made to **Results**, **Outcomes**, Paragraph Two:

The DY and CR also did not significantly differ between the chewing gum and control groups (*P* = 0.224 and *P* = 0.114, respectively).

In the original article, there was a mistake in Figures 2, 3 as published. The order of Figures 2, 3 are reversed. The corrected Figures 2, 3 appears below.

**Figure 2:**
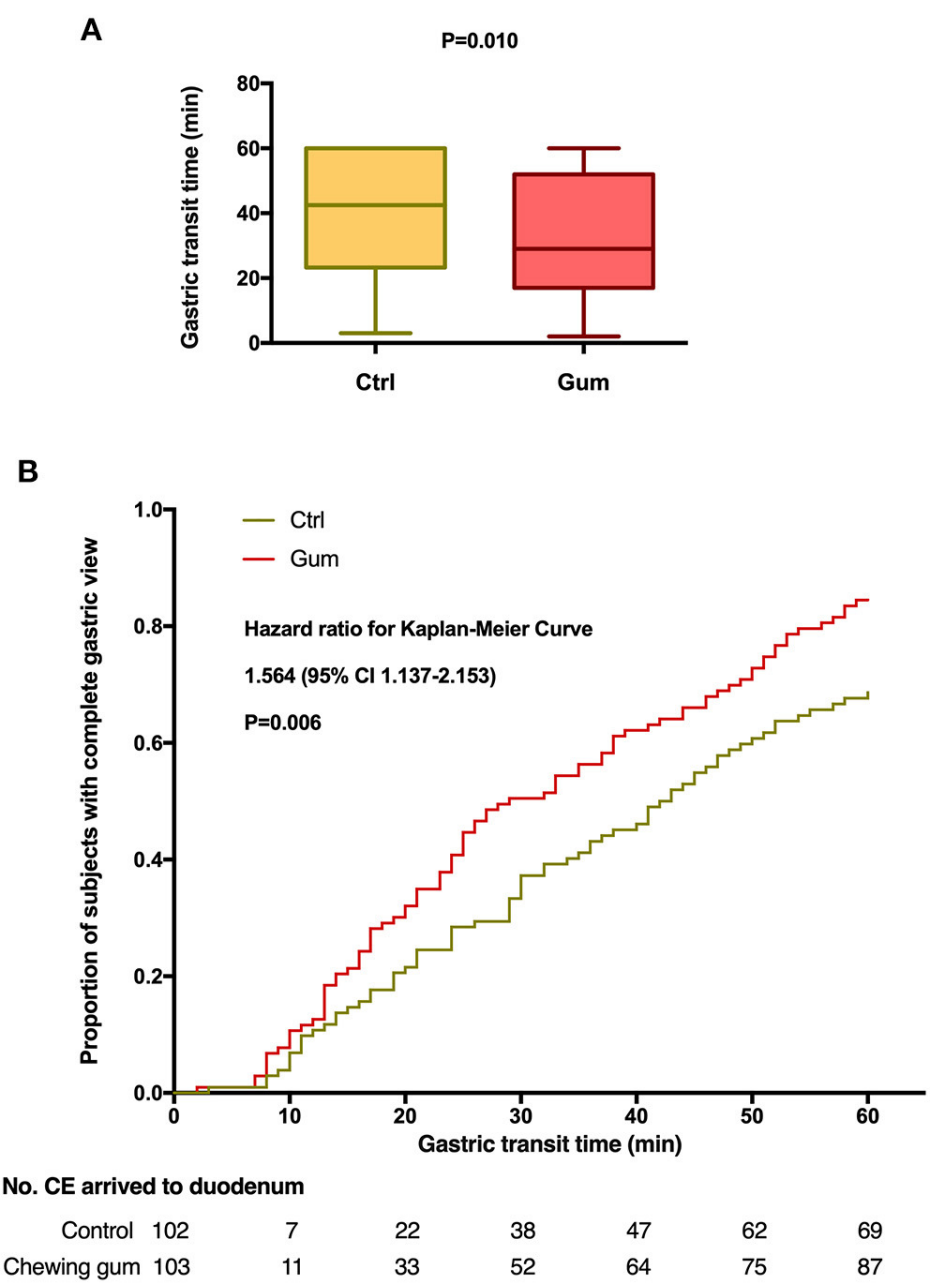
Gastric transit time during capsule endoscopy in the chewing gum group and control group. **(A)** A boxplot with medians and quartiles; **(B)** Kaplan–Meier curves for time to complete gastric view.

**Figure 3:**
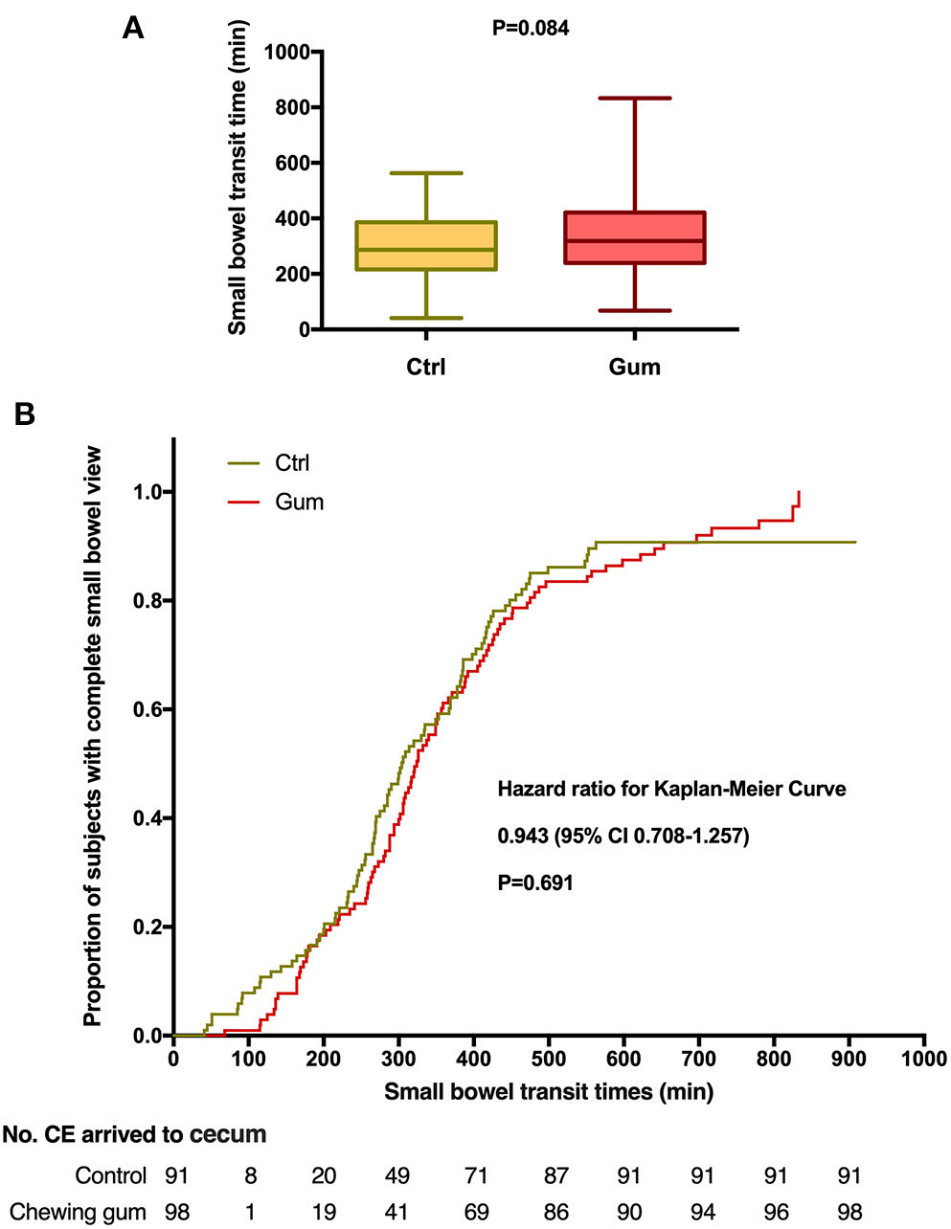
Small bowel transit time during capsule endoscopy in the chewing gum group and control group. **(A)** A boxplot with medians and quartiles; **(B)** Kaplan–Meier curves for time to complete small bowel view.

The authors apologize for this error and state that this does not change the scientific conclusions of the article in any way. The original article has been updated.

## Publisher's Note

All claims expressed in this article are solely those of the authors and do not necessarily represent those of their affiliated organizations, or those of the publisher, the editors and the reviewers. Any product that may be evaluated in this article, or claim that may be made by its manufacturer, is not guaranteed or endorsed by the publisher.

